# A systematic review of the efficacy of cancer vaccines in advanced breast cancer

**DOI:** 10.1007/s12282-025-01751-1

**Published:** 2025-08-05

**Authors:** Anna Charalampopoulou, Charalampos Filippatos, Panagiotis Malandrakis, Flora Zagouri, Maria Gavriatopoulou, Ioannis Ntanasis-Stathopoulos

**Affiliations:** Department of Clinical Therapeutics, Athens School of Medicine, National and Kapodistrian University of Athens, Athens, Greece

**Keywords:** Breast cancer, Vaccines, Immunotherapy, Advanced

## Abstract

**Background:**

Cancer vaccines offer an innovative approach to treating advanced breast cancer, by activating the immune system to recognize and target cancer cells, aiming to boost immune response, reduce relapse, and enhance patients’ quality of life.

**Methods:**

A systematic database search was conducted for peer-reviewed, full-text articles on clinical trials focused on cancer vaccines for advanced breast cancer, in the MEDLINE, SCOPUS, Cochrane and ClinicalTrials.gov databases, from conception until October 23, 2024.

**Results:**

A total of 19 trials, involving 747 patients with advanced breast cancer, were included in this systematic review. Cancer vaccine trials rely primarily on immunological endpoints, with initial small-scale studies showing modest efficacy improvements and highly variable immune response rates (44–100%), suggesting opportunities for further optimization and broader clinical application.

**Conclusion:**

While the overall quality of the studies was acceptable, heterogeneity in results and limited survival or progression outcomes prevented data synthesis. In conclusion, despite promising findings, the lack of large-scale randomized trials limits definitive conclusions on cancer vaccine efficacy, highlighting the need for Phase III trials to confirm their clinical utility and long-term impact.

**Supplementary Information:**

The online version contains supplementary material available at 10.1007/s12282-025-01751-1.

## Introduction

Breast cancer is the most commonly diagnosed cancer in women worldwide, accounting for 23.8% of all female cancer cases, according to GLOBOCAN 2022. It remains the leading cause of cancer-related deaths in females, with over 660,000 fatalities reported in 2022 alone [1, 2]. Breast cancer (BC) is a highly heterogeneous neoplasm with distinct molecular subtypes which can be classified as Luminal A or B, human epidermal growth factor receptor 2 (HER2) positive, or triple negative breast cancer (TNBC) based on the immunohistochemical expression of hormone receptors, such as the expression of estrogen receptors (ER +), progesterone receptors (PR +), human epidermal growth factor receptors (HER2 +), grade, and proliferation index (Ki-67) [3–5]. Although standard treatments, such as surgery, radiotherapy, and chemotherapy, effectively target primary tumors, cancer recurrence remains common; immunotherapy addresses this with techniques like immune checkpoint inhibitors (ICIs), CAR T-cell therapy, bispecific monoclonal antibodies (mabs) and cancer vaccines [6]. Cancer immunotherapy aims to overcome tumor recurrence by reprogramming the body’s immune system to recognize cancer-specific antigens and the tumors producing them, targeting cancer cells for destruction [7]. Cancer vaccines offer a promising solution by stimulating long-term immune memory, enabling the immune system to recognize and attack multiple tumor antigens. Therefore, developing effective BC vaccines is crucial for reducing recurrence and improving patient outcomes [8, 9].

In the recent years, the clinical development of immunotherapy has accelerated, particularly with the introduction of ICIs for TNBC, sparking an increase in clinical trials exploring immunotherapeutic strategies across different BC subtypes [10]. Furthermore, findings of a recent systematic review indicate that immunotherapy, whether used alone or alongside standard cancer treatments, holds significant promise for boosting overall survival (OS) and progression-free survival (PFS) in patients, particularly those with recurrent disease after initial treatments [11]. Clinical trials also reveal that immunotherapy typically causes fewer and more manageable side effects than treatments like chemotherapy, which often result in higher adverse effects, especially in older patients [12]. Despite early clinical trials showing promising results, no BC vaccine has demonstrated significant therapeutic benefits in Phase III studies [13]. The current challenges include short-lived immune responses, immune tolerance to tumor antigens, and limited understanding of the underlying mechanisms of action [14].

The purpose of this systematic review is to provide a comprehensive overview of the clinical trials focused on novel vaccines for the neoadjuvant and/or adjuvant treatment of advanced and metastatic BC (MBC), emphasizing on the efficacy of therapeutic active immunization strategies and discussing the potential role and prospects of vaccines in the treatment of advanced BC.

## Materials and methods

### Study eligibility criteria

Study designs such as randomized controlled trials (RCTs) and nonrandomized clinical trials were included to further assess the efficacy of vaccination either as a stand-alone treatment or in combination with any of the typical cancer treatments used. Ongoing studies with available results from interim analyses and completed clinical trials were also considered eligible. Terminated or withdrawn clinical trials and those from ClinicalTrials.gov without reported outcomes are excluded to prevent bias and maintain the integrity of the evidence synthesis. The study focused on Phase I/II, II and III clinical trials. Phase I studies were not included in the review because they primarily assess safety, tolerability and dosing rather than efficacy. Any articles that included preclinical studies, case reports or series, observational studies, retrospective studies, systematic reviews, or meta-analyses were also excluded. Cancer vaccine studies performed in patients with advanced BC that did not report efficacy results, were also excluded.

### Search strategy

This systematic review was designed and performed according to the Preferred Reporting Items for Systematic Reviews and Meta-Analyses (PRISMA) guidelines. The systematic search was conducted up to October 23rd, 2024 in MEDLINE (PUBMED), SCOPUS, Cochrane Central Register of Controlled Trials and ClinicalTrials.gov. The algorithm implemented was the following: (breast AND (advanced OR “late stage” OR “stage IV” OR “stage III” OR metastatic OR progressive) AND (cancer OR cancers OR neoplasm OR carcinoma OR carcinomas OR neoplasia OR malignancy OR malignancies OR malignant)) AND (“breast cancer vaccine” OR “therapeutic vaccine” OR “vaccination” OR “active immunotherapy” OR “active immunization”) AND (Safety OR Efficacy). Additionally, in the advanced search in ClinicalTrials.gov, breast cancer was selected in the “Condition of disease” field, interventional studies were selected in the “Study type” field and cancer vaccine, vaccine therapy was selected in the “Intervention/treatment” field. The following keywords were used in the “Other terms” to select relevant breast cancer clinical trials: cancer vaccine, therapeutic vaccine, vaccine. In case of identifying both interim and final analyses for the same trial, the final results were preferred. Only trials with results published in the last 5 years were included, as to ensure up to date information is used, especially considering that vaccination in BC is a novel clinical approach.

### Data abstraction

For each of the eligible studies, the following data were collected from ClinicalTrials.gov: clinical trial identifier number (NCT number), BC subtype and stage, name of the vaccine, sample size, study design, phase and primary and secondary endpoints. As for the databases MEDLINE (PUBMED), SCOPUS, Cochrane Central Register of Controlled Trials the following data were collected: first author and publication year, eligibility criteria regarding BC subtype and stage, name of the vaccine, sample size, study design, and primary and secondary endpoints.

### Risk of bias assessment

Risk of bias was assessed for all included studies using two tools, based on study design. The Cochrane Risk of Bias Tool for Randomized Trials (RoB 2) was applied to randomized trials, while the ROBINS-I tool was used for nonrandomized and single-arm studies, with appropriate adaptations for the latter.

## Results

### Overview of clinical trials of vaccines in advanced-metastatic breast cancer

**Fig. 1:**
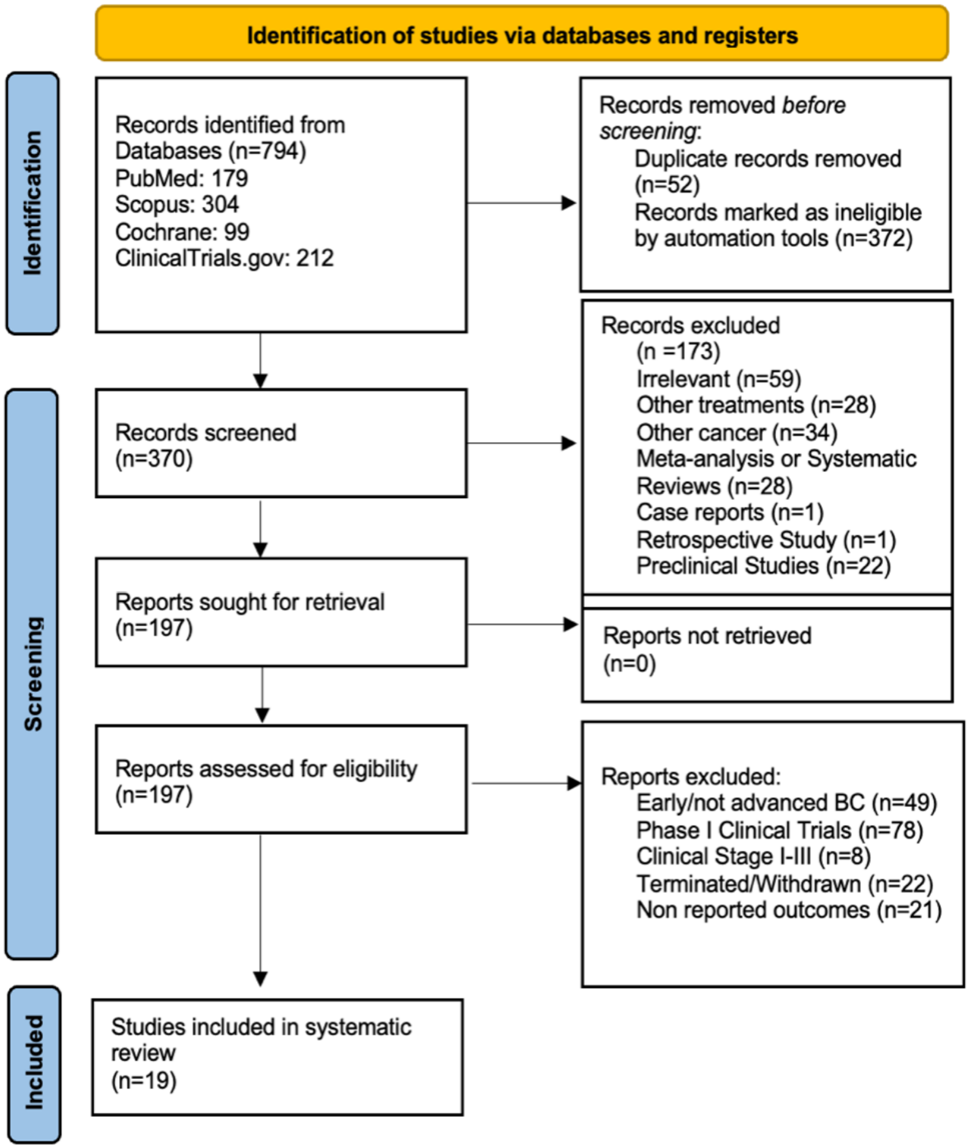
Flow diagram of search strategy according to PRISMA 2020 guidelines

Our systematic search retrieved 794 records: 179 from MEDLINE (PUBMED), 304 from SCOPUS, 99 from the Cochrane Central Register of Controlled Trials, and 212 from ClinicalTrials.gov. Automated tools were used in MEDLINE, SCOPUS, and Cochrane databases (English language, last 5 years), excluding 372 ineligible studies. Αn additional 52 records were marked as duplicate. Thus, 370 records (titles and abstracts) were screened. Of these, 173 were excluded for reasons such as irrelevance (*n* = 59), different interventions (*n* = 28), other cancer types (*n* = 34), meta-analyses/systematic reviews (*n* = 28), case reports (n = 1), retrospective studies (*n* = 1), and preclinical studies (*n* = 22). The remaining 197 records underwent full-text review, leading to the exclusion of 178: early-stage BC (*n* = 49), Phase I trials (*n* = 78), unspecified advanced stages (*n* = 8), terminated/withdrawn studies (*n* = 22), and no outcomes reported (*n* = 21). Nineteen trials met the inclusion criteria for final analysis. The steps of this screening process are portrayed in the following PRISMA 2020 flow diagram (Fig. 1).

Of the 19 eligible trials, 10 were Phase I/II, 9 were Phase II, and none were Phase III. Most (90%, 17/19) were open-label, with 57% (11/19) using single-group allocation and only 5 being randomized. Nine studies focused on advanced or MBC, 8 on HER2 + MBC, and 2 on metastatic TNBC. Table 1 summarizes trial categories and primary efficacy endpoints. Regarding cancer vaccine types, 6 trials evaluated dendritic cell-based vaccines, 5 peptide-based, 4 whole cell-based, 3 viral vector-based, and 1 carbohydrate-based.

**Table 1:** Overview of clinical trials included

Clinical trials (*N* = 19)	Number of trials	No of trials with available efficacy results as primary outcome
Trial phase		
Phase I/II	10	2
Phase II	9	6
Cancer type/stage		
Triple negative BC	2	–
Metastatic/locally advanced	9	6
HER2 +	8	2
Allocation		
Randomized	6	3
Nonrandomized	2	1
Single arm	11	4
Blinding		
Open label	17	7
Double blind	2	1

### Phase I/II clinical trials with cancer vaccines in advanced/metastatic breast cancer

#### Peptide-based BC vaccines

Among the 19 eligible trials, Phase I/II studies of peptide vaccines were common (21%, 4/19), all involving HER2 + BC and none as monotherapy. Main characteristics are presented in Table 2. Two studies, Stanton et al., and Disis et al., evaluated the HER2/neu peptide vaccine in open-label, single-group designs with approximately 20 patients each [15, 16]. In Disis et al., the vaccine was combined with trastuzumab, while Stanton et al. added cyclophosphamide, sargramostim, and HER2-specific T cells. Both had toxicity as the primary endpoint, with Disis also measuring immune response using ELISpot and HLA-A2 analysis, showing significant T cell activation in > 70% of patients. OS in Disis showed almost 50% survival at 5 years. Stanton reported immunological endpoints with 60% response and one evaluable patient showing a complete PET response [17].

**Table 2:** Phase I/II clinical trial data extraction categorized by vaccine type

Study	Type/stage of BC	Vaccine	N	Design	Phase	Primary endpoint	Secondary endpoints	Prior line of treatment
Peptide-based vaccines								
NCT01922921	HER2 + Stage IV BC^1^	HER2 ICD^4^ Peptide-Based Vaccine	31	Single-center, Double -blind, Parallel assignment, randomized	I/II	Toxicity	Induction of IFN-gamma and CD107a Expression in NK Cells^10^ via Flow Cytometry	At least 1 ChT^21^ & continue anti-HER2 therapy per standard of care
Stanton et al. [15]	HER2 + Stage IV BC	HER2/neu peptide vaccine	23	Single-center, Open-label, Single arm	I/II	Toxicity	T-cell persistence post-immunization assessment	NI^22^
Disis et al. [16]	HER2 + , Stage IV BC	HER-2/neu peptide vaccine	22	Single-center, Open-label, Single arm	I/II	Immune response, Number of AE^7^	OS^11^	At least 1
NCT00952692	HER2 + , MBC^2^	dHER2^5^	12	Single-center, Open-label, Single arm	I/II	Safety, Immune Response	CR^12^, PR^13^, TTP^14^	NI
Whole cell-based vaccines								
Wiseman et al. [18]	recurrent and/or MBC	SV-BR-1-GM	24	Multicentered, Open-label, Single arm	I/II	Safety	Safety, OTR^15^, Rate of Nonprogression of Tumors, TR^16^	At least 1 ChT
Dendritic cell-based vaccines								
Soliman et al. [19]	MBC	Ad.p53 DC^6^ Vaccine	39	Single-center, Open-label, Single arm	I/II	Phase I: MTD^8^, Phase II: SD^9^	Phase I: OR^17^ Phase II: PFS^18^, Clinical Response	Up to 2 prior lines of ChT for Phase II
NCT00082641	stage III BC overexp. p53	Ad. p53 DC Vaccine	24	Single-center, Open-label, Parallel assignment randomized	I/II	Toxicity, Immune response	–	NI
Zhang et al. [20]	stage III—IV BC	DC vaccine	4 out of 10	Single-center, Open-label, Single arm	I/II	Safety	Clinical Response	NI
NCT02018458	LA TNBC^3^	DC vaccine	10	Single-center, Parallel assignment, Open-label, Nonrandomized	I/II	Safety	pCR^19^, DFS^20^	No treatment the last 5 years
Viral vector-based vaccines								
NCT00088413	MBC	PANVAC	12	Single-center, Open-label, Nonrandomized	I/II	Clinical Response, AE	–	At least 1 failed therapy (66.7% had ≥ 3 regimens of ChT)

^1^*BC* breast cancer, ^2^*MBC* metastatic breast cancer, ^3^*LA TNBC* locally advanced metastatic breast cancer, ^4^*HER2 ICD* human epidermal growth factor receptor 2 intracellular domain, ^5^*dHER2* truncated recombinant HER2/neu peptide, ^6^*Ad. p53 DC* Adenovirus p53 Dendritic Cell, ^7^*AE* Adverse events, ^8^*MTD* maximum tolerated dose, ^9^*SD* stable disease, ^10^ *IFN-gamma and CD107a Expression in NK Cells* interferon-gamma and cluster of differentiation 107a in natural killers cells, ^11^*OS* overall survival, ^12^*CR* complete response, ^13^*PR* partial response, ^14^*TTP* time to progression, ^15^*OTR* objective tumor regression, ^16^*TR* tumor response, ^17^*OR* objective response, ^8^*PFS*: progression free survival, ^9^*pCR* pathological clinical response, ^20^*DFS* disease free survival, ^21^*ChT* chemotherapy, ^22^*NI* no information

NCT00952692 followed the same design and assessed HER2 + AS15 combined with lapatinib in 12 patients. Safety was confirmed, but only 8.3% showed a T cell response, with no Complete Response (CR), Partial Response (PR) observed.

NCT01922921 was a double-blind, randomized trial (*n* = 31). Both arms (1:1) received HER2 ICD peptide-based vaccines plus trastuzumab ± pertuzumab. The experimental arm also received polysaccharide-K, showing higher IFN-gamma and CD107a responses compared to the control. OS and PFS were not reported.

#### Whole cell-based breast cancer vaccines

Wiseman et al., conducted an open-label, single-arm, multicenter trial on advanced, treatment-resistant MBC [18]. The intervention involved SV-BR-1-GM, with low-dose cyclophosphamide before vaccination and interferon alpha afterward. Tumor response (CR, PR, SD) was assessed using Response Evaluation Criteria in Solid Tumors (RECIST) and immune-related RECIST (iRECIST) criteria, along with PFS, immune response and quality of life. Among 26 patients, 23 received one cycle, 21 completed two, and 17 completed three. No Objective Response Rate (ORR), defined as CR or PR was observed, but 16.7% achieved Stable Disease (SD), with a median response duration of 105.5 days (95% CI 79–197).

#### Dendritic cell-based breast cancer vaccines

Four trials assessed the safety and efficacy of dendritic cell (DC)-based vaccines in BC. Soliman et al., conducted a single-center, open-label study of adenovirus-p53-transduced DC (Ad.p53-DC) vaccine plus 1-methyl-D-tryptophan (1-MT) in ER + / − , HER2 − MBC patients with p53 overexpression [19]. Of 44 patients enrolled, 39 received at least one vaccine dose. In Phase II, 19% achieved SD, while 47% had Clinical Response (CR, PR, or SD). Median PFS and OS were 13.3 (95% CI 12.97–21.85) weeks and 20.7 weeks 95% CI 25.75–46.15), respectively. Nine patients who responded to salvage chemotherapy post-trial had a median OS of 69.4 weeks (95% CI 30.1–122.1).

NCT00082641 was a randomized trial evaluating p53-infected autologous DCs in women with Stage III BC. Participants received either neoadjuvant or adjuvant chemotherapy and adjuvant radiotherapy. Vaccination schedules differed between two arms. Immune response reached 100% in the first arm and 53% in the second, indicating schedule-dependent immunogenicity.

Zhang et al., studied WT1 peptide-pulsed DC vaccines in 10 patients with advanced BC, ovarian, or gastric cancer [20]. The WT1 gene encodes a zinc finger transcription factor that is overexpressed in numerous hematological malignancies and solid tumors [21]. Among the 4 BC patients, all achieved SD after initial treatment. After completing WT1-pulsed DC vaccination, 2 patients achieved PR, while 2 maintained SD.

NCT02018458 is a parallel assignment nonrandomized trial, combined cyclin B1/WT1/CEF antigen-loaded DC vaccination with preoperative chemotherapy. Of 10 eligible patients, only those with locally advanced TNBC enrolled. The study reported 40% pathological Clinical Response (pCR) and Disease Free Survival (DFS) of 15.6 months (95% CI 6.5–26.3). No ER + /HER2 − patients were enrolled.

#### Viral vector-based breast cancer vaccines

NCT00088413 evaluated the clinical response of PANVAC-V (priming) and PANVAC-F (boosting) vaccines in 51 patients, including 12 with advanced BC. The vaccines target Carcinoembryonic Antigen (CEA) and Mucin 1 (MUC-1), proteins commonly overexpressed in adenocarcinomas, and were combined with sargramostim (GM-CSF) to enhance immune response without significant toxicity [22, 23]. This open-label, nonrandomized trial included three experimental cohorts. Patients received PANVAC-V on day 1, followed by three PANVAC-F doses and sargramostim at the injection site for 4 days after each vaccination. Responders could receive monthly or quarterly boosts if stable. Clinical response, assessed by RECIST over 6 months, showed that one patient (8.3%) achieved CR, four (33.3%) had SD, and seven (58.3%) experienced PD.

### Phase II clinical trials with cancer vaccines in advanced/metastatic breast cancer

#### Peptide-based breast cancer vaccines

Peptide-based vaccines offer a promising approach for advanced cancers but their therapeutic efficacy remains unproven in clinical settings and insufficient for regulatory approval [24]. Toh et al., conducted a monocentric, open-label, single-arm Phase II trial with 14 patients with refractory metastatic TNBC [25]. The vaccine contained 19 peptides. The primary outcome was safety, while secondary outcomes included PFS and peptide-specific immune induction. Main characteristics are presented in Table 3. Clinical responses per RECIST showed six patients with SD and eight with PD. Median PFS was 1.5 months (95% CI 1.0–15.6) and OS was 11.5 months (95% CI 1.5–42.1). Among 10 patients who completed the trial, PFS and OS were 5.8 (95% CI 1.4–18.9) months and 24.0 months (95% CI 2.3‐not reached), respectively. Those who did not complete the study had significantly worse outcomes, with a median PFS of 0.9 months (95% CI 0.5–1.2) and OS of 1.4 months (95% CI 1.0–6.0). The differences between groups were statistically significant.

**Table 3:** Phase II clinical trial data extraction categorized by vaccine type

Study	BC Type/stage	Vaccine	N	Design	Phase	Primary endpoint	Secondary endpoints	Prior line of treatment
Peptide-based vaccines								
Toh et al. [25]	mTNBC^1^	mixed 19‐peptide vaccine	14	Single-center, Open-label, Single arm	II	Safety^4^	PFS, Clinical Response	At least 1 (71% < 3 regiments of ChT^11^ | 21% ≥ 3 regimens of ChT)
Whole cell-based vaccines								
NCT00971737	MBC^2^ (NOT HER-2 +)	Vaccine made from gene-modified tumor	63	Single-center, Open-label, Parallel assignment, randomized	II	Immune response, Number of AE^7^	OS^11^	NI^22^
Chen et al. [26]	HER2 + , Stage IV BC^3^	allogeneic GM-CSF^4^-secreting whole cell BC vaccine	22	Single-center, Open-label, Single arm	II	Safety, Immune Response	CR^12^, PR^13^, TTP^14^	NI
NCT00847171	HER2 + , MBC	Allogeneic GM-CSF-Secreting Breast Tumor Vaccine	20	Single-center, Open-label, Single arm	II	Safety	Clinical Benefit	Any number of prior ChT allowed
Dendritic cell-based vaccines								
Vincent et al., 2023	HER2 + , MBC	Multi-epitope autologous DC^5^ vaccine	17	Single-center, Open-label, Single arm	II	Number of Patients With Response	–	NI
NCT00499083	Stage II/​III BC	Autologous DC vaccine	17	Multicentered, Open -label, Single arm	II	pCR^8^	–	No prior chemotherapy or radiotherapy
Viral vector-based vaccines								
Crosby et al. [27]	HER2 + , advanced BC	VRP-HER2^6^ Vaccine	9	Single-center, Open-label, Parallel assignment, randomized	II	Number of Participants With a Positive T Cell Response	AE, Clinical response rate	At least 1
Heery et al. [28]	MBC	PANVAC	48	Multicentered, Open -label, Cross over assignment, randomized	II	PFS	OS	At least 1
Carbohydrate-based vaccines								
Huang et al. [29]	MBC	Globo H-KLH vaccine adagloxad simolenin (OBI-822)/OBI-821	349	Multicentered, Double-blind, randomized	II	Clinical Response, AE	–	At least 1 failed therapy (66.7% had ≥ 3 regimens of ChT)

^1^*mTNBC* metastatic triple negative brest cancer, ^2^*MBC* metastatic breast cancer, ^3^*BC* breast cancer, ^4^*GM-CSF* granulocyte–macrophage colony stimulating, ^5^*DC* dendritic cell, ^6^*VRP-HER2* virus-like replicon particles of human epidermal growth factor receptor 2, ^7^*AE* adverse event, ^8^*pCR* pathological complete response, ^9^*PFS* progressive free survival, ^10^*OS* overall survival, ^12^*ChT* chemotherapy,^13^ *NI* no information

#### Whole cell-based breast cancer vaccines

Cytokine-modified tumor cell vaccines that secrete GM-CSF can induce robust T cell-dependent immunity. Multiple clinical studies have demonstrated their single agent safety and bioactivity in cancer patients, but their therapeutic impact remains unproven probably because vaccination alone is not potent enough to induce immune responses sufficiently [13].

NCT00971737 evaluated cyclophosphamide with an allogeneic GM-CSF-secreting breast tumor vaccine, with or without trastuzumab, for HER2 negative MBC. It was an open-label, randomized trial with two arms. The control group received cyclophosphamide and the vaccine, while the experimental group also received trastuzumab. The primary endpoint was Clinical Benefit (CB) measured by PFS at six months. Of the 60 randomized patients, 33% (95% CI 17–53) in the control group and 37% (95% CI 20–56) in the experimental group achieved CB at six months, with no significant difference between arms.

Two additional trials focused on HER2 + MBC with similar single-arm, open-label designs combining trastuzumab, cyclophosphamide, and an allogeneic GM-CSF-secreting breast tumor vaccine. The Chen et al., study reported CB at six months of 55% (11/20; 95% CI 32–77%; *p* = 0.013) and 40% at one year [26]. Median PFS was 7 months (95% CI 4–16) and OS was 42 months (95% CI 22–70), with a five-year survival rate of 30%. In NCT00847171, a similar regimen was used, but only 7 of the 20 enrolled patients had confirmed MBC. Among these, 6 demonstrated no evidence of disease progression in a frame of 4 years, yielding a PFS rate of 85.7%.

#### Viral vector-based breast cancer vaccines

The NCT03632941 trial, presented as Crosby et al., in Table 3, was an open-label, parallel assignment study with three cohorts: VRP-HER2 immunization, pembrolizumab, and a combination of both [27]. The primary endpoint was a positive T-cell response by ELISpot over five years. The VRP-HER2 arm (*n* = 1) achieved a 100% response rate, the pembrolizumab arm (*n* = 2) had a 50% response rate, and the combination arm (*n* = 5) showed a 60% response rate. All arms reported a 100% SD response rate, except for one participant missing data in both the VRP-HER2 and pembrolizumab groups.

Heery et al., evaluated docetaxel with PAN-VAC in a multicenter, open-label, randomized trial. In the first arm (*n* = 23), patients received docetaxel and dexamethasone alone. In the second arm (*n* = 25), docetaxel was combined with PAN-VAC and sargramostim [28]. The median PFS was 3.8 months (95% CI 2.6–8.4) in the first arm and 6.6 months (95% CI: 3.7–9.4) in the combination arm, showing improved PFS with PAN-VAC.

#### Carbohydrate-based breast cancer vaccines

Huang et al., conducted a multicenter, randomized, placebo-controlled, double-blind trial enrolling 349 women with MBC who had achieved CB after at least one prior line of therapy [29]. Participants were randomized 2:1 to receive AS/OBI-821 (*n* = 225) or placebo (*n* = 124), both with cyclophosphamide. The primary endpoint was investigator-assessed PFS. Median PFS was 7.6 months (95% CI 6.5–10.9) in the AS/OBI-821 group versus 9.2 months (95% CI 7.3–11.3) in the placebo group (HR 0.96, *p* = 0.77). Median OS was not reached in either group. The results indicated that the disease progression occurred in 71% of patients in the experimental group and 73% of those in the placebo group. Disease progression was the primary cause of treatment discontinuation. Median OS was not reached in either treatment group by the conclusion of the trial.

### Overview of clinical trial efficacy results using cancer vaccines in advanced breast cancer

The clinical results of studies included in this review are summarized in Table 4, which categorizes them based on the type of cancer vaccine. Of the 19 studies included in the review, there is significant variability in the endpoints used to assess efficacy, regardless of whether efficacy was a primary or secondary outcome. Specifically, the different endpoints reported are as follows: Number of participants with SD, Clinical Response, CB, Number of participants with Response, Number of participants with Immune Response, pCR, PFS and OS. The variability in endpoints reflects the complex nature of cancer vaccines, the diversity of BC subtypes, the stages of treatment development, and the different aims of the studies. This necessitates the use of multiple assessment methods to fully understand their efficacy.

**Table 4:** Summary of clinical results for cancer vaccine studies, by vaccine type

Study	BC type/stage	Primary efficacy endpoint	Secondary efficacy endpoints
Peptide-based cancer vaccines			
NCT01922921	HER2 + ^1^ Stage IV BC^2^	–	Induction of IFN-gamma and CD107a Expression in NK Cells^13^ via Flow Cytometry: 69.2% > = twofold increase
Stanton et al. [15]	HER2 + Stage IV BC	–	No of patients with T cell response^*^: 78.9% Response of bone-only disease by FDG-PET^14^: 100.0% | ^*^up to 2 years following the last infusion
Disis et al. [16]	HER2 + Stage IV BC	Immune Response up to 1.5 years: HLA-2: 73.7% ELIspot: 84.2%	OS up to 5 years: 47.6%
NCT00952692	HER2 + MBC^3^	–	OCR^15^ (CR or PR) in 26 weeks: 0.0% Time to progression: 55 days (95% CI: 41 to 188)
Toh et al. [25]	mTNBC^4^	–	PFS: 1.5 months (95% CI, 1.0‐15.6) OS^16^: 24 months (95% CI, 2.3‐not reached) Clinical Response: 43.0% SD, 57.0% PD^17^
Whole cell-based breast cancer vaccines			
Wiseman et al. [18]	recurrent and/or metastatic lesions BC	–	OTR^18^(CR or PR)*: 0.0% Rate of Nonprogression of Tumors (CR, PR, SD)^*^: 16.7% Durability of Tumor Response*: 105.5 days (95% CI:79–197) | *an average of 1 year
NCT00971737	MBC	CB^6^ as assessed by PFS^7*^ Arm I: 33.0% (95% CI, 17–53) without transtazumab Arm II: 37.0% (95% CI, 20–56) with transtazumab |^*^ at 6 months post-intervention	–
Chen et al. [26]	HER2 + Stage IV BC	CB at 6 months: 55.0% (95%CI:32–77) at 1 year: 40.0% (95% CI:19–64)	PFS: 7 months (95% CI:4–16) OS: 42 months (95% CI: 22–70) 5-year survival rate was 30.0% (95% CI:12–54)
NCT00847171	HER2 + MBC	–	CB assessed by PFS: 85.7%
Dendritic cell-based breast cancer vaccines			
Soliman, et al. [19]	MBC	*Phase II*: No of participants with SD^8^ up to 16 weeks: 19.0%	*Phase I*: ORR^19^: 0.0% *Phase II*: Clinical Response Rate up to 3 years: 47.0% (5% CR, 33.0% PR, 9.5% SD) PFS up to 3 years: 6.85 weeks (95% CI: 3.8–18.1) OS: 20.71 weeks
NCT00082641	Stage III BC overexp. p53	No of patients with Immune Response: Arm I: 100.0% Arm II: 53.0%	–
Zhang et al. [20]	Stage III-IV BC	–	Clinical Response: 50.0% PR, 50.0% SD
NCT02018458	LA TNBC^5^	–	pCR^*^ in a year 40.0% RCB1^*20^: 30.0% | ^*^ in 1 year DFS^21^ in 36 months:15.6 months (95%CI:6.5–26.32)
Vincent et al., 2023	HER2 + MBC	No of participants with Response up to 5–6 years (CR^9^,PR^10^,OR^11^): 0.0%	–
NCT00499083	Stage II/III BC	pCR^12^ at definitive surgery: 14.0%	–
Viral Vector-based breast cancer vaccines			
NCT00088413	MBC	Clinical Response up to 6 months: 8.3% CR^4^, 0.0% PR^5^, 33.3% SD, 58.3% PD^6^	–
Crosby et al. [27]	HER2 + advanced BC	No of participants with positive T cell Response Arm I: 100.0% Arm II: 50.0% Arm III: 60.0%	Overall Response (SD) up to 5 years: 100%
Heery et al. [28]	MBC	PFS at 19.7 months: Arm I: 7.9 months Arm II: 3.9 months (HR_ΙvsII_ = 0.65, 95% CI: 0.34 – 1.14)	–
Carbohydrate-based breast cancer vaccine			
Huang et al. [29]	MBC	PFS: 7.6 months (95% CI: 6.5–10.9)	OS: no results

^1^*HER2 +*  human epiderman growth factor receptor 2 positive, ^2^*BC* breast cancer, ^3^*MBC* metastatic breast cancer, ^4^*mTNBC* metastatic triple negative breast cancer, ^5^*LA TNBC* locally advanced triple negative breast cancer, ^6^*CB* clinical benefi, ^7^*PFS* progression free survival, ^8^*SD* stable disease, ^9^*CR* complete response, ^10^PR partial response, ^11^*OR* overall response, ^12^*pCR* pathological complete response, ^13^*IFN gamma and CD107a Expression in NK Cells* Interferon-gamma and cluster of differentiation 107a in natural killers cells, ^14^*FDG-PET* fluorodeoxyglucose positron emission tomography, ^15^*OCR* objective clinical response, ^16^*OS* overall survival, ^17^*PD* progressive disease, ^18^*OTR* objective tumor response, ^19^*ORR* objective response rate, ^20^*RCB1* residual cancer burden 1, ^21^*DFS* disease free survival

The results were not favorable for some studies. In the study by Vincent et al., 2023, no patients responded to treatment and in NCT00088413 more than half of the patients experienced disease progression (58.3%). In addition, there are studies that showed that combining a cancer vaccine with a standard of care has no effect on its efficacy, such as in the NCT00971737 study, where the CB assessed by PFS at 6 months was 33% (95%CI 17–53) when patients received only cyclophosphamide and vaccine and 37% (95%CI 20–56) when patients received cyclophosphamide, vaccine and trastuzumab.

On the other hand, NCT00082641 showed that patients who received neoadjuvant or adjuvant chemotherapy in combination with the vaccine all exhibited an immune response, in contrast to patients who received the vaccine after adjuvant radiation therapy, where 53% of patients exhibited an immune response. In addition, there were studies, such as Heery et al. [28] Huang et al. [29] and Chen et al. [26], that reported a median PFS of approximately 7 months, with Huang et al. [29] showing the most promising PFS result of 7.6 months (95% CI 6.5–10.9) in stage IV BC. Also, in the Crosby et al. [27] study, the overall response (SD) reached 100% up to 5 years and in the Disis et al. [16] study, OS up to 5 years was 47.6%.

Although efficacy was not always the primary endpoint in the studies included in the review, most of them, 15 out of 19, focused primarily on safety or immunogenicity. In these studies, the primary endpoint was primarily the safety and toxicity of the treatment, rather than the efficacy in cancer treatment response. Most of these trials (7/8) were Phase I/II and one early Phase II as they emphasize the primary endpoints of safety and immune responses to demonstrate that the vaccine is safe and works by targeting the immune system. Efficacy is included as a secondary endpoint to examine early signs of CB, and the results presented in Table 4 are particularly promising: in the Zhang et al., [20] study, the clinical response showed 50% PR and 50% SD. In addition, in the NCT00847171 study, CB assessed by PFS was 85.7%. These findings highlight the potential efficacy of vaccines and the value of diverse endpoints in their evaluation.

### Overview of clinical trial safety results using cancer vaccines in advanced breast cancer

The majority of reported Adverse Events (AEs) were only of Grade 1 or 2, data for AEs is reported descriptively in Table 5. Local complications at the injection site such as erythema or pruritus were most reported, experienced by more than half of the patients in every trial. Fever was also a commonly reported AE, while other AEs included fatigue, nausea, diarrhea, anemia or myalgia. Overall, the vaccine products were well tolerated. Most of the serious adverse events (SAEs) reported in the studies were attributed to the concomitant therapy rather than the vaccine. The AEs primarily associated with the vaccine included local reactions such as injection site reactions, extravasation changes, allergic reactions, skin infections, and urticaria. Among the 19 studies, only 3 reported patient discontinuation due to AE. In Wiseman et al. [18] 30.8% of patients enrolled in the study discontinued due to AE, with most being unrelated to the treatment except for cancer pain (probably related) and GERD (possibly related). In Vincent et al., 2023, 11.7% of patients were removed from the study due to allergic reactions (Grade 2). Finally, in Huang et al. [29] treatment discontinuation due to AEs was less than 1%. None of the 22 studies that were terminated or withdrawn were due to AE or safety concerns; the primary reasons were the lack of patient recruitment (no patients) or unavailable funding.

**Table 5:** Summary of safety results for cancer vaccine studies, by vaccine type

Name of study	Percentage of patients experiencing any AE		Percentage of patients experiencing Grade 3 + AE
	Total	Local complications (injection)	No of SAE
Peptide-based vaccines			
NCT01922921	100.00%	86.00%	0.00%
Stanton et al. [15]	100.00%	95.47%	4.35%
Disis et al. [16]	100.00%	95.24%	14.29%
NCT00952692	91.67%	50.00%	16.67%
Toh et al. [25]	NI	64.20%	NI
Whole-cell based vaccines			
Wiseman et al. [18]	100.00%	45.83%	33.30%
NCT00971737	100.00%	100.00%	0.00%
Chen et al. [26]	100.00%	73.91%	5.00%
NCT00847171	100.00%	100.00%	0.00%
Dendritic cell-based vaccines			
Soliman et al. [19]	90.24%	NI	34.15%
NCT00082641	54.55%	NI	ARM I: 45.45% ARM II: 8.33%
Zhang et al. [20]	NI	NI	NI
NCT02018458	100.00%	NI	30.00%
Vincent et al., 2023	70.59%	NI	11.76%
NCT00499083	100.00%	NI	33.33%
Viral vector-based vaccines			
Crosby et al. [27]	100.00%	100.00%	ARM I (vaccine): 0.00% ARM III (vaccine + ICI): 20.00%
Heery et al. [28]	92.00%	68.00%	12.00%
NCT00088413	100.00%	84.60%	100.00% (BC + ovarian)
Carbohydrate-based vaccines			
Huang et al. [29]	98.20%	77.20%	12.90%

### Risk of bias assessment

Among the six randomized trials, three provided full-text results. Two of them were rated as having “some concerns” due to missing outcome data and unclear pre-specification of outcomes and analyses. One trial was judged as high risk because of substantial missing data without sufficient justification. For the remaining 13 nonrandomized and single-arm studies, two were assessed as low risk and seven as moderate risk, mainly due to limited information in protocols or registries, especially regarding selective reporting. The ROBINS-I tool was applied only to relevant domains, as certain aspects are not applicable to single-arm designs. Detailed results are presented in the Supplementary Figs. 1 and 2.

## Discussion

Breast cancer represents the third most studied tumor type for cancer vaccination, following melanoma and cervical cancer, yet there is no FDA-approved vaccine for BC at any stage; early, advanced, or metastatic [30]. This stands in contrast to other cancers, such as melanoma, where the FDA recently granted accelerated approval for lifileucel, an autologous T cell immunotherapy, and cervical cancer, which benefits from three licensed prophylactic HPV vaccines (Gardasil, Gardasil 9, and Cervarix) [31, 32]. Most clinical trials on BC vaccines have focused on the metastatic setting, where the tumor microenvironment is likely affected by inhibitory mechanisms [33]. Vaccine monotherapy in precancerous or adjuvant settings and combinations with ICIs in advanced stages are considered promising strategies. However, progress in BC vaccine development remains slow [34, 35]. In 2021, only 2.3% of the registered clinical trials for BC were experimental Phase III trials, while the remaining 97.7% were early-Phase studies. In fact, a 20 year systematic review revealed no significant improvement in ORR with vaccine-based approaches [36]. Phase-III trials have failed to show benefits in either metastatic or early-stage BC. Notably, the NP-S vaccine with GM-CSF showed no improvement in three-year DFS, leading to the early termination of the trial [37]. Similarly, HER2-targeted vaccines, such as AE37, also failed to demonstrate a significant increase in DFS [37, 38]. However, after two decades of setbacks, BC vaccines have regained interest due to technological advancements and the momentum generated by the COVID-19 vaccination campaign [39]. Emerging platforms such as gene based, lipid nanoparticles, virus-like particles, and polymeric and nondegradable nanoparticles are evolving as more efficient and immunogenic delivery vehicles, offering renewed hope for future breakthroughs [40].

In this systematic review, among 19 included clinical trials, 747 patients with advanced or metastatic BC received at least one inoculation. Cancer vaccines trials rely heavily on immunological endpoints to evaluate vaccine-induced immune responses and their potential link to clinical efficacy. Although these endpoints are essential for evaluating immune activation, they do not always correlate consistently with clinical outcomes such as OS or PFS. This limits the interpretation of immune responses as surrogate markers of CB and underscores the need for more rigorous trials that incorporate long-term efficacy endpoints. Initial small-scale trials have shown modest improvements in vaccine efficacy, highlighting opportunities for further optimization and broader clinical application. Notably, the studies such as NCT01922921, NCT00082641, Disis et al., [16] Crosby et al. [27] and Stanton et al. [15] have shown that vaccines induced an immune response in over 65% of patients with HER2 + BC. This may be attributed to the higher immunogenicity of HER2 + tumors compared to luminal types, as HER2 + cancers are associated with increased rates of cell proliferation and genomic instability [41, 42]. The predominance of peptide-based vaccines in these studies (60%) may reflect the presence of highly immunogenic epitopes in HER2 + tumors, which facilitates the development of appropriate adjuvants and targeted delivery systems crucial for peptide vaccine efficacy [43, 44].

One of the key strengths of our study is its comprehensive approach, as it includes all subtypes of advanced and metastatic BC, providing a broader perspective compared to existing reviews. A recent systematic review focused solely on TNBC across all stages, primarily assessing vaccine safety and efficacy, whereas we focus exclusively on advanced-stage disease across all BC subtypes [45]. Similarly, another systematic review and meta-analysis narrowed its scope to HER2 peptide vaccines [46]. Furthermore, one other systematic and meta-analysis exclusively studied the E75 vaccine in early-stage breast cancer, while our study focuses on all vaccines used in advanced BC [47]. Finally, a recent 20 year systematic review and meta-analysis limits its data sources to PubMed and applies stringent inclusion criteria, requiring studies to report specific clinical outcomes such as ORR, PFS, or OS [36]. By contrast, our study draws from multiple databases and does not restrict inclusion based on specific clinical endpoints, thus offering a more comprehensive and representative analysis of the field.

The current clinical trials of vaccines for advanced and metastatic BC face significant challenges, with limited improvements in treatment outcomes. A key limitation of this systematic review is the absence of RCTs and the heterogeneity of study populations, which hinders direct comparisons and prevents data synthesis—thus limiting the generalizability of findings. Most included studies were early-phase (Phase I/II), single-arm trials with small cohorts, lacking comparison groups, thus increasing the risk of bias. No Phase III trials in advanced BC populations are currently registered in ClinicalTrials.gov, as vaccines are generally more effective in early-stage disease with less compromised immune systems [48]. Notably, large-scale Phase III trials, such as the GLORIA trial (NCT03562637), are already underway in early-stage BC [49]. Patient selection poses an additional limitation. In total, 568 out of 747 patients had received at least one prior line of chemotherapy (as shown in Tables 2 and 3), with several being heavily pretreated. Most trials required patients to have completed previous therapies within 14 or 28 days before enrollment, and cancer vaccines were typically administered in combination with ongoing treatments, such as endocrine therapy, trastuzumab, or cyclophosphamide. This heavily pretreated population may exhibit reduced immune competence, potentially limiting vaccine efficacy. Factors such as host immunocompetence, immunosuppressive tumor microenvironment, and timing of vaccination should be carefully considered in future trial designs. These factors highlight the complexity of cancer vaccine trials and the need for well-designed, randomized studies in more homogeneous populations.

Despite promising immune responses in some studies, several trials reported no CB. In NCT00952692 and Vincent et al., 2023, no patients achieved PR or CR, and in NCT00088413, over half of the patients experienced disease progression (58.3%). Similarly, the Wiseman et al., 2024 study reported no ORR, with only 16.7% achieving SD. In other cases, the addition of a vaccine to standard therapies, as in NCT00971737, yielded minimal improvement in PFS. These findings underscore the need for deeper exploration into the mechanisms of vaccine resistance or failure, including patient-specific immune suppression, antigen selection, and suboptimal combination strategies.

Based on these considerations, the proposed study design emphasizes the necessity for large scale, randomized Phase III Clinical Trials, aimed at optimizing delivery strategies, adjuvant selection and the development of personalized neoantigen-based vaccines. Careful evaluation of safety and the use of clinically meaningful endpoints—such as OS, PFS, and ORR—are essential for accurately assessing therapeutic efficacy. For future research, additional experimental approaches are recommended, including immunophenotyping pre/post vaccination to longitudinally profile the immune landscape (e.g., TILs, Treg cells, and myeloid-derived suppressor cells) and identify predictors of response. Furthermore, a biomarker-driven trial design, incorporating markers such as HER2, p53, or WT1, could facilitate patient stratification and improve treatment outcomes. Finally, combination trials exploring the synergistic effects of cancer vaccines with checkpoint inhibitors are of particular relevance in immune-silent tumors, such as ER + /HER2 − breast cancer.

## Conclusions

There is a need for larger, well-designed Phase III trials to comprehensively evaluate the therapeutic potential of cancer vaccines. Such studies will be essential to determine whether these vaccines can offer meaningful CB, particularly in combination with other therapies or in specific subgroups of patients.

## Supplementary Information

Below is the link to the electronic supplementary material.

Supplementary file1 (DOCX 132 KB)

## Data Availability

Further data available upon request from the corresponding author.
