# Supplementary material for: A systematic review of the efficacy of cancer vaccines in advanced breast cancer
Source: Breast Cancer. 2025 Aug 5;32(5):892–904. doi: 10.1007/s12282-025-01751-1 (PMC12394260; doi:10.1007/s12282-025-01751-1)
Supplement: Supplementary file 1 — Supplementary file1 (DOCX 132 KB) [file 12282_2025_1751_MOESM1_ESM.docx]

**PICOS**

The clinical questions of the current systematic review are based on the following **PICOS**:

**P**opulation: advanced/metastatic BC (Stage III-IV)

**I**ntervention: cancer vaccine

**C**omparator: patients who have not been vaccinated

**O**utcome: efficacy

**S**tudy type: clinical trials

**RISK OF BIAS ASESSMENT**

| **Unique ID** | **Outcome** | **Weight** | **D1** | **D2** | **D3** | **D4** | **D5** | **Overall** |  |  |  |  |  |
| --- | --- | --- | --- | --- | --- | --- | --- | --- | --- | --- | --- | --- | --- |
| Chiun-Sheng Huang, 2020 | PFS | NA |  |  |  |  |  |  |  |  |  |  |  |
| Christopher R Heery, 2015 | PFS | NA |  |  |  |  |  |  |  |  |  |  |  |
| NCT01922921 | Immune Response | NA |  |  |  |  |  |  |  |  |  |  |  |


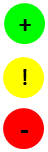


Low risk

Some concerns

High risk

**Figure 1:**  Traffic light plot for risk of bias assessment on all completed trials using the Cochrane risk-of-bias tool for randomized trials (RoB 2).

| **Unique ID** | **Outcome** | **Weight** | **D1** | **D2** | **D3** | **D4** | **D5 D6 D7** | **Overall** |  |  |  |  |  |  |
| --- | --- | --- | --- | --- | --- | --- | --- | --- | --- | --- | --- | --- | --- | --- |
| Charles L Wiseman, 2024 | CR, PR, SD | NA |  | NA  NA  NA  NA  NA  NA  NA  NA  NA  NA |  |  | NA  NA  NA  NA  NA  NA  NA  NA  NA  NA |  |  |  |  |  |  |  |
| Gang Chen, 2014 | CB | NA |  |  |  |  |  |  |  |  |  |  |  |  |
| Hatem Soliman, 2018 | SD | NA |  |  |  |  |  |  |  |  |  |  |  |  |
| Benjamin G Vincent, 2023 | CR,PR, OR | NA |  |  |  |  |  |  |  |  |  |  |  |  |
| Wen Zhang, 2019 | RR | NA |  |  |  |  |  |  |  |  |  |  |  |  |
| Uhi Toh, 2020 | PFS | NA |  |  |  |  |  |  |  |  |  |  |  |  |
| Mary L Disis, 2009 | Immune Response | NA |  |  |  |  |  |  |  |  |  |  |  |  |
| NCT00952692 | CR, PR | NA |  |  |  |  |  |  |  |  |  |  |  |  |
| NCT02018458 | pCR | NA |  |  |  |  |  |  |  |  |  |  |  |  |
| NCT00088413 | CR, PR | NA |  | NA |  |  |  |  |  |  |  |  |  |  |


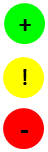


Low risk

Moderate risk

Serious risk

Critical risk

**Figure 2:** Traffic light plot for risk of bias assessment on all completed trials using the Robins-I tool for single-arm and non-randomized trials
